# LDL-cholesterol lowering effect of a new dietary supplement: an open label, controlled, randomized, cross-over clinical trial in patients with mild-to-moderate hypercholesterolemia

**DOI:** 10.1186/s12944-018-0775-8

**Published:** 2018-05-24

**Authors:** S. Magno, G. Ceccarini, C. Pelosini, R. Jaccheri, J. Vitti, P. Fierabracci, G. Salvetti, G. Airoldi, M. Minale, G. Saponati, F. Santini

**Affiliations:** 1 Obesity Center at the Endocrinology Unit, University Hospital of Pisa, Via Paradisa 2, Pisa, Italy; 2 Studio Associato Airoldi Cicogna Ghirri, Via Manzoni 40, Milan, Italy; 3 ISPharm srl, Via Oberdan 43, Lucca, Italy

**Keywords:** Hypercholesterolemia, LDL-cholesterol (LDL-C), Triglycerides, Red reast rice, Monacolin K

## Abstract

**Background:**

Hypercholesterolemia is a major risk factor for cardiovascular disorders and requires specific intervention through an adequate lifestyle (diet and physical exercise) and, if necessary, an appropriate drug treatment. Lipid-lowering drugs, although generally efficacious, may sometimes cause adverse events. A growing attention has been devoted to the correction of dyslipidemias through the use of dietary supplements. The aim of this study was to assess the lipid-lowering activity and safety of a dietary supplement containing monacolin K, L-arginine, coenzyme Q10 and ascorbic acid, named Argicolina (A), compared to a commercially available product containing monacolin K and coenzyme Q10, Normolip 5 (N).

**Methods:**

This was a single center, controlled, randomized, open-label, cross-over clinical study enrolling 20 Caucasian outpatients aged 18–75 years with serum LDL-C between 130 and 180 mg/dL. Patients assumed two different dietary supplements (A and N) both containing monacolin K 10 mg for 8 weeks each, separated by a 4-week wash-out period. Evaluated parameters were: Total cholesterol (Tot-C), low density lipoprotein cholesterol (LDL-C), high density lipoprotein cholesterol (HDL-C), triglycerides (TG), fasting blood glucose, aspartate aminotransferase, alanine aminotransferase, creatinekinase, gamma-glutamyl-transpeptidase, brachial arterial pressure and heart rate, measured at the start and at the end of each treatment period. Safety was monitored through the study.

**Results:**

LDL-C decreased by 23.3% during treatment with N (*p* < 0.0001) and by 25.6% during treatment with A (p < 0.0001); the LDL-C mean reduction was 36.4 (95% CI: 45,6–27,1) mg/dL during N treatment and 40.1 (95% CI: 49.2–30,9) mg/dL during A treatment. Tot-C decreased significantly (p < 0.0001) within each treatment period. HDL-C increase was negligible during A whereas it was significant during N. TG diminished markedly during A and not significantly during N. The difference between treatments was not statistically significant for all variables. No serious or severe adverse events occurred during the study.

**Conclusions:**

Our results confirm the clinically meaningful LDL-C lowering properties of monacolin K. At variance with a supplement already in the market (N), the novel association (A) of monacolin K with L-arginine, coenzime Q10 and ascorbic acid also produces a significant reduction of triglycerides without significant effects on HDL.

**Trial registration:**

ClinicalTrials.gov ID: NCT03425630.

## Background

Cardiovascular diseases (CVDs) rank first as the current leading cause of death [1]. Identification, prevention and management of the risk factors for CVDs are therefore a priority in public healthcare programs [2]. Among CVDs risk factors, one of the most important is dyslipidemia, primarily hypercholesterolemia, which may be corrected through an adequate lifestyle (diet and physical exercise) and, if necessary, an appropriate drug treatment [3]. The results achieved with lipid-lowering drugs, however, are not always satisfactory, and untoward effects (mainly myalgia and myopathies) may sometimes emerge that cause patients to discontinue the treatment [4, 5]. Over the past few years a growing attention has been devoted to the correction of dyslipidemias through the use of dietary supplements, either because some patients may have milder forms of hypercholesterolemia or as an alternative to statins in patients who may have experienced side effects, although the potential adverse effects caused by supplements have not been fully investigated. There is evidence of a relationship between some food constituents and a reduction in CVD incidence [6–8]. Monacolin K is a substance obtained during rice fermentation by the fungus *Monascus purpureus*, used for thousands of years in China to produce rice wine. Fermented rice owes its red color (hence the name “red yeast rice”) to various pigments produced by the fungus, including monascorubramin and rubropunctamin. In 1979 Akira Endo of the Tokyo University isolated a metabolite produced by the fungus with a strong inhibitory activity toward the enzyme HMG-CoA reductase (3-hydroxy-3-methyl-glutaryl coenzyme A reductase), which mediates the step from hydroxymethylglutaryl coenzyme A to mevalonic acid early in the pathway to the endogenous synthesis of cholesterol [9]. This metabolite was later characterised as a member of the monacolin group and identified as monacolin K, a structural analogue of lovastatin. Several clinical studies have demonstrated a lipid-lowering effect of monacolin K, alone [10, 11] or in association with other compounds [12]. Studies have shown a reduction in serum total cholesterol (Tot-C) between 12 and 30% after the administration of products containing from 3 to 10 mg of monacolin K, for treatments ranging from 4 weeks up to 1 year [11, 13–15]. Some of these trials have reported a lower incidence of myopathies compared to statins [16]. A recent metanalysis on products containing monacolin K have shown that the expected mean reduction, compared to placebo, was 37.5 mg/dL (95%CI, 30.9–43.7; *p* < 0.00001) for Tot-C and 33.6 mg/dL (95%CI, 27.5–39.8; *p* < 0.00001) for LDL-cholesterol (LDL-C) [17]. A novel dietary supplement named Argicolina (A) containing monacolin K, L-arginine, coenzyme Q10 and ascorbic acid has been developed by Damor Pharmaceuticals (Naples, Italy). L-arginine is an essential aminoacid, a substrate in the synthesis of nitric oxide (NO) catalysed by the NO-synthetase expressed in the vessel endothelium, therefore acting as a vasodilator and an inhibitor of platelet aggregation [18–23]. Experimental work showed that the association of L-arginine with a statin increased NO production by endothelial cells compared to statin alone [24]. Coenzyme Q has antioxidant activity and is widely used in dietary supplements for subjects with raised lipid levels or cardiovascular risk [25]. Ascorbic acid has antioxidant and vasoprotective activity [26, 27]. The diverse and complementary properties of the components of A suggest that this product may be useful in treating subjects with hyperlipidemia or cardiovascular risk. The aim of this study was to assess the lipid-lowering activity and safety of the proposed formulation in patients with mild-moderate hypercholesterolemia, compared to a commercially available dietary supplement containing monacolin K and coenzyme Q10 (Normolip 5: N) (ESI - Albissola Marina, Savona, Italy). The study was conducted according to a randomized cross-over design.

## Methods

### Patients selection

Between July 2016 and April 2017 eligible patients were recruited among the outpatients attending the Obesity Center of the Endocrinology Unit 1, Cisanello Hospital, Pisa, Italy. Patients aged 18–75 years with serum LDL-C between 130 and 180 mg/dL, not significantly modified by an appropriate dietetic regimen were considered eligible for the study. Exclusion criteria were: pregnancy or breast-feeding; known liver, renal or muscle diseases; serum triglycerides (TG) greater than 350 mg/dL; previous cardiovascular events; concomitant neoplastic or immunodepressive diseases; use of lipid-lowering drugs or dietary supplements within the last 3 weeks; concurrent use of thiazide diuretics, oral contraceptives containing estrogen or progestogen, systemic corticosteroids; use of psycho-active substances, drug or alcohol abuse; neurological or psychiatric diseases that could affect consent validity or impair the patient’s adherence to the study protocol. Thirty patients, all Caucasian, were screened. Ten were excluded during the screening process because they did not fulfill all the inclusion criteria (screening failure). Twenty patients were thus randomized, 10 to the A > N sequence and 10 to the N > A sequence.

### Study design

**Fig. 1:**
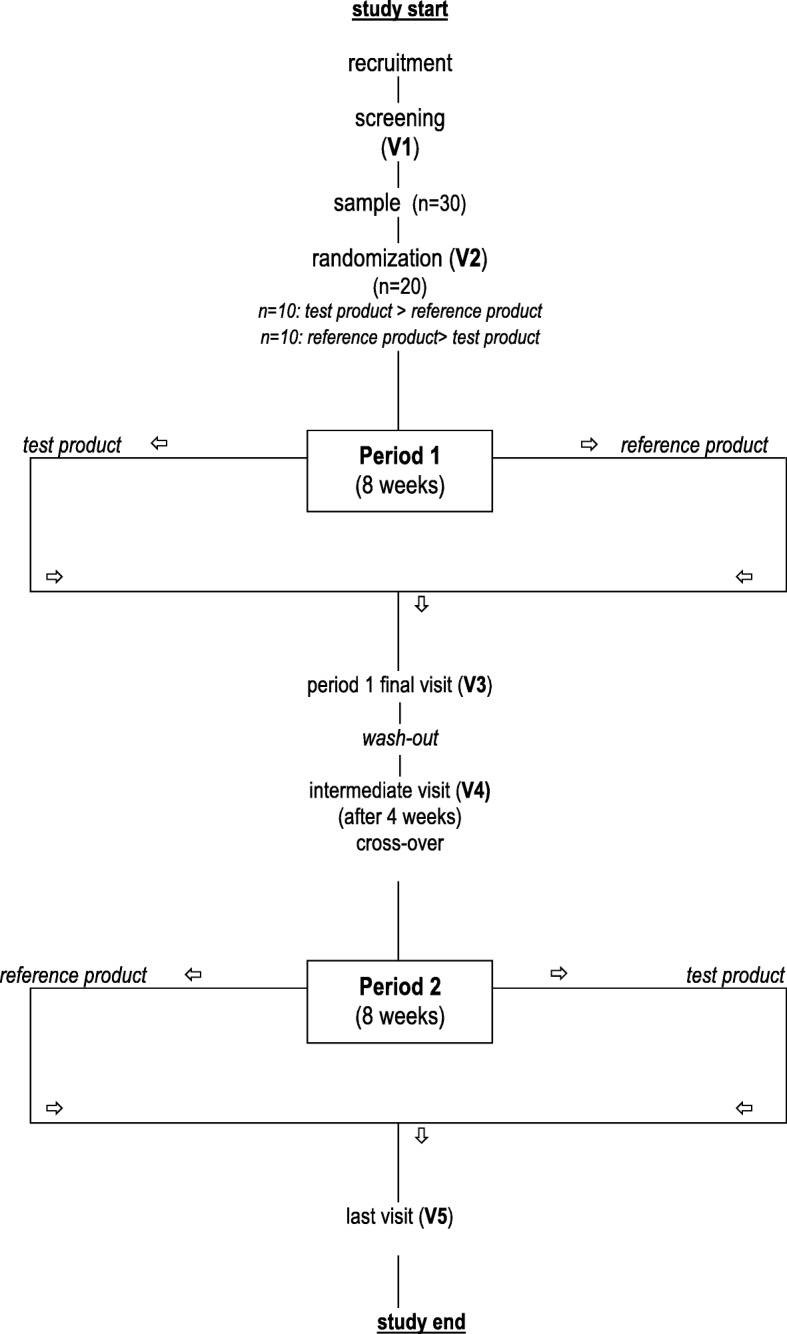
Study flow-chart

The study was conducted in a single center according to a controlled, randomized, open-label, cross-over design. Each patient had to assume, in a randomized sequence, both treatments (A, 1 sachet/day; N, 1 capsule/day) for 8 weeks each separated by a 4-week wash-out period. The study plan included the initial screening visit (V1), an entry visit at start of the first treatment period (V2), a visit at the end of the first treatment period (V3, 56 ± 5 days after V2), a wash-out period of 4 weeks (±5 days), a visit at start of the second (crossed over) treatment period (V4), and a visit at the end of the second treatment period (V5, 56 ± 5 days after V4) (Fig. 1). Tot-C, LDL-C, HDL-cholesterol (HDL-C), TG, fasting blood glucose, aspartate aminotransferase (AST), alanine aminotransferase (ALT), creatinekinase (CK), gamma-glutamyl-transpeptidase (GGT), brachial arterial pressure and heart rate were measured at V1, V3, V4 and V5. Blood analyses were centrally performed in the laboratory of the Endocrinology Unit using standard enzymatic techniques; LDL-C was directly measured. Clinical safety was monitored throughout the study. If required, the patient could be re-evaluated at any time during the study, aside of the visits scheduled.

### Statistical methods

The minimum level of statistical significance was set to *p* < 0.05 two-sided, therefore 95% confidence limits (95%CIs) were calculated. All reported *p*-values and CIs are two-sided.

The primary efficacy variable was the LDL-C change between the start and the end of each treatment period, expressed as a percentage of the initial value. Therefore, mean and 95%CIs of changes within treatment periods (from V2 to V3 and from V4 to V5) for the experimental and the control treatment, irrespective of sequence, were calculated. The main analysis was the determination of the two-sided 95%CI of the between-treatment mean difference in the primary variable, computationally analogous to a paired t test. Setting 0.10 (i.e. 10% of the initial value) as the minimum clinically relevant difference, the two treatments were considered equivalent if the two-sided 95%CI of the difference in their LDL-C change from baseline was entirely between − 0.10 and + 0.10. Parallel calculations were carried out on absolute, rather than relative to baseline, LDL-C changes. Tot-C and HDL-C were analyzed as described above for LDL-C; for TG levels (which were approximately log-normally distributed) analogous calculations were performed on logarithmic transformations and changes were expressed as ratios. Between-treatment comparisons were expressed as A − N differences for cholesterol values and as A/N ratios for TG values. The effects on LDL-C were additionally tested in sensitivity multivariate analyses: variance for cross-over studies on final-baseline changes adjusting for period effects, and analysis of covariance on the difference between the final values adjusted for sequence and for the difference between the baseline values. Efficacy analyses had to be performed in the intention-to-treat population, i.e. all patients with at least one post-baseline control. A sensitivity analysis of the primary variable was also planned in the per-protocol population, i.e. all patients without major protocol violations. Safety results had to be reported in all patients who had assumed at least one dose of one study drug. Statistical analyses were performed by the Studio Associato Airoldi Cicogna and Ghirri, Milan, using the SAS Software version 9.4 (SAS Inc., Cary, NC).

### Sample size

The sample size was calculated for the main efficacy analysis described above, i.e. the determination of the two-sided 95%CI of the between-treatment mean difference in the LDL-C change from baseline. Assuming a standard deviation (SD) of the difference no greater than 0.12, based on a previous cross-over study for the difference between monacolin K and placebo [11], it was estimated that 18 patients were required to prove the equivalence with a power of 0.80. This figure was rounded to 20 enrolled patients allowing for possible exclusions from the analysis.

## Results

### Patients characteristics and compliance

A patient was lost to follow-up after V2 (study entry) and was therefore excluded for analysis. No major violations of the study protocol occurred in the other 19 patients. Therefore the intention-to-treat, per-protocol and safety populations were constituted by the same patients. The demographic characteristics of the 19 patients included in the analysis are shown in Table 1.

**Table 1:** Characteristics of the patients at entry

	all patients (19 patients)	sequence N-A (10 patients)	sequence A-N (9 patients)
Gender (F/M)	13/6	7/3	6/3
Age (years)	54.7 ± 9.3 (36–70)	56.2 ± 9.6 (37–69)	53.1 ± 9.2 (36–70)
Height (cm)	168 ± 11	169 ± 9	167 ± 13
Weight (Kg)	86 ± 24	84 ± 24	87 ± 26
Body Mass Index (Kg/m^2^)	30.3 ± 8.2	29.4 ± 8.2	31.3 ± 8.6

Data are expressed as mean ± SD (min-max range)

The baseline characteristics of the patients assigned to the two different sequences of treatment (A > N or N > A) were similar, only slight differences being observed regarding the parameters studied. Ten patients were affected by one or more metabolic or endocrine diseases: obesity (*n* = 9), hypothyroidism treated with L-thyroxine (*n* = 5), and type-2 diabetes mellitus treated with metformin (*n* = 2). Six patients were on antihypertensive therapy. The ratio between the number of doses presumably assumed (products delivered minus returned) and the treatment duration in days (equivalent to the number of doses to be taken as per the scheduled one-daily dose regimen), a measure of treatment compliance, was greater than 90% in all 19 patients who completed the study.

### Efficacy

**Fig. 2:**
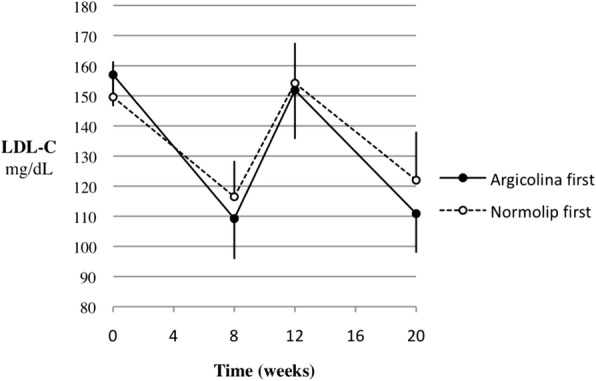
LDL-C values (mean and SD) through the study, split by treatment sequence

**Fig. 3:**
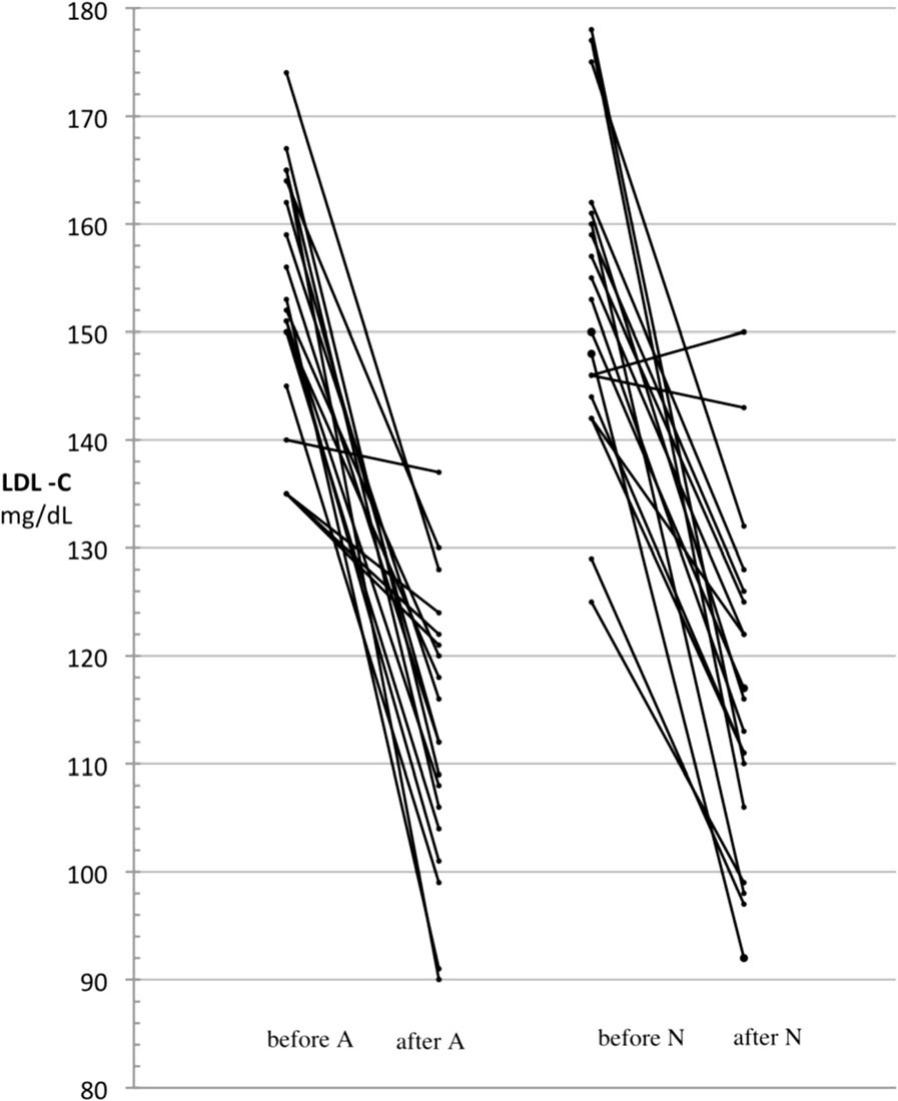
Individual values of LDL cholesterol (LDL-C, mg/dL) before and after Argicolina (A) and Normolip (N) treatment

LDL-C values and their changes during the study are reported in Table 2. LDL-C decreased by 23.3% during treatment with N and by 25.6% during treatment with A. The A − N difference, i.e. the mean difference between the within-period changes observed with A and N, was − 2.3% (the minus sign meaning a greater reduction during treatment with A compared to N). The 95%CI of this difference was between − 7.4 and + 2.8%, entirely within the interval of − 10 to + 10% defined as clinical equivalence and used in sample size calculation. In parallel, the absolute (i.e., not referred to the initial value) LDL-C mean reduction was 36.4 mg/dL during N treatment and 40.1 mg/dL during A treatment, the mean A − N difference being − 3.7 mg/dL (95%CI, − 13.0 to + 5.6). LDL-C reductions within each treatment, either as absolute values or expressed as ratio of the initial value, were highly significant (*p* < 0.0001). The results of the analysis of variance adjusting for period effects on final−baseline LDL-C changes were very similar to those of the main analysis above. The mean A − N difference relative to baseline was − 2.3% (95%CI, − 7.4 to + 2.9; *p* = 0.36), with *p* = 0.70 for the period effect. The mean absolute A − N difference was − 3.8 mg/dL (95%CI, − 13.4 to + 5.7; *p* = 0.41), with *p* = 0.52 for the period effect. The analysis of covariance on the difference between the final values adjusted for sequence and for the difference between the baseline values yielded a mean A − N absolute difference of − 3.6 mg/dL, very close to the other estimates reported above, but with a narrower 95%CI (− 9.7 to + 2.5; *p* = 0.23), with *p* = 0.49 for the sequence effect and *p* = 0.83 for the difference between the baseline values. No carry-over effect was observed, based on the fact that plasma LDL-C levels at the end of the wash-out were similar to pre-study values (Fig. 2). Individual values at start and end of each treatment showed a decrease of LDL-C in all patients, except for one who changed from 146 to 150 mg/dL during the first-period treatment with N (Fig. 3). Irrespective of treatment period, decrease from abnormal (≥ 130 mg/dL) to normal LDL-C values was obtained in 17 of 19 patients during A treatment (89%) and in 14 of 17 during N treatment (82%; 2 patients were still in the normal range after the wash-out and remained so at the end of treatment). Tot-C, HDL-C and TG values measured before and after each treatment and their differences are reported in Tables 3, 4 and 5.

**Table 2:** LDL-C (mg/dL) in the 19 patients who completed the study

	Argicolina	Normolip	Argicolina − Normolip
At start of treatment	153.1 ± 11.6	153.1 ± 14.4	0.0 (− 7.3 +7.3)
At end of treatment	113.1 ± 12.8 *	116.7 ± 15.4 *	− 3.7 (−9.5 +2.1)
Δ Final–Initial	−40.1 (−49.2 −30.9)	−36.4 (−45.6 −27.1)	−3.7 (−13.0 +5.6)
% Δ(Final–Initial) / Initial	−25.6 (−31.1 –20.1)	−23.3 (−28.7 −17.9)	−2.3 (−7.4 +2.8)

Data are expressed as mean ± SD, (95%CI), and analyzed by Student’s t test for paired data (end vs. start of treatment), * *p* < 0.0001

**Table 3:** Total-C (mg/dL) in the 19 patients who completed the study

	Argicolina	Normolip	Argicolina−Normolip
At start of treatment	221.1 ± 16.5	221.6 ± 18.0	−0.5 (−9.8 +8.8)
At end of treatment	178.3 ± 16.3*	187.0 ± 17.1*	−8.7 (− 15.3 −2.1)
Δ Final–Initial	−42.7 (−53.6 −31.8)	−34.6 (−46.1 −23.1)	−8.2 (−19.9 +3.5)
% Δ (Final–Initial) / Initial	−19.0 (−23.5 −14.4)	−15.2 (−19.8 −10.5)	−3.8 (−8.2 +0.6)

Data are expressed as mean ± SD, (95%CI), and analyzed by Student’s t test for paired data (end vs. start of treatment), * *p* < 0.0001

**Table 4:** HDL-C (mg/dL) in the 19 patients who completed the study

	Argicolina	Normolip	Argicolina − Normolip
At start of treatment	56.3 ± 15.6	56.6 ± 16.0	−0.3 (−2.9 +2.3)
At end of treatment	57.7 ± 15.3	59.2 ± 17.1*	−1.5 (−3.5 +0.5)
Δ Final–Initial	+1.4 (−0.7 +3.5)	+2.6 (+0.8 +4.3)	−1.2 (−3.8 +1.4)
% Δ(Final–Initial) / Initial	+3.0 (−1.3 +7.3)	+4.3 (+1.5 +7.1)	−1.3 (−6.0 +3.3)

Data are expressed as mean ± SD, (95%CI), and analyzed by Student’s t test for paired data (end vs. start of treatment), * *p* < 0.01

**Table 5:** Triglycerides (mg/dL) in the 19 patients who completed the study

	Argicolina	Normolip	Argicolina/Normolip rate
At start of treatment	104.2	97.6	106.8 (96.3118.4)
At end of treatment	86.9*	93.1	93.3 (81.0107.4)
Final / Initial %	83.4 (72.0 96.5)	95.4 (85.9106.0)	87.4 (73.3104.1)

Data are expressed as geometric mean (95% CI) and analyzed by Student’s t test for paired data (end vs. start of treatment) after logarithmic transformation, * *p* < 0.05

Like LDL-C, Tot-C decrease within each treatment period was always highly significant (*p* < 0.0001), while the between-treatment comparison showed a slightly, non-significantly greater reduction with A, both as absolute value (− 8.2 mg/dL; *p* = 0.16) and as ratio of baseline (− 3.8%; *p* = 0.086).

**Fig. 4:**
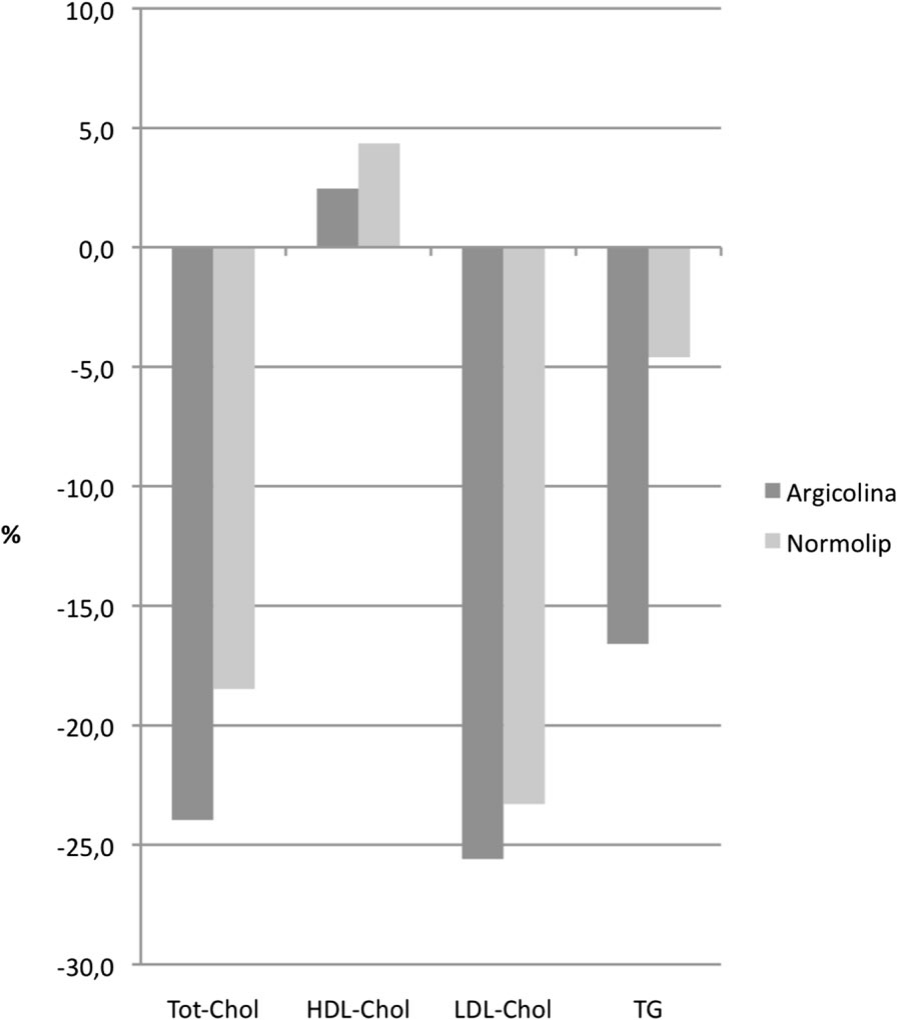
Percent changes during A and N treatments of total cholesterol, HDL-cholesterol LDL-cholesterol and triglycerides (TG). Data are expressed as (final-initial) / initial values

HDL-C increase was negligible during A and significant during N (Table 4), whereas TG diminished markedly during A and not significantly during N (Table 5); the difference between treatments, however, was not statistically significant for both variables (*p* > 0.30 and *p* = 0.12, respectively). The percent changes of the efficacy variables from start to end of each treatment are summarized in Fig. 4.

### Safety

No serious or severe adverse events occurred during the study; no event required treatment interruption or remedial therapy. Moderate and transient gastrointestinal disturbances occurred in three patients, two receiving N (constipation and flatulence) and one receiving A (diarrhea). Other moderate and transient adverse events referred by one patient each were eczema and headache during treatment with N and myalgia during treatment with A. Serum CK levels above the upper limit of normal (190 U/L) were observed in three patients: one after treatment with A (268 U/L) –previously not treated with N, one after both A and N (276 and 246 U/L, respectively), and one after the wash-out period between A and N (320 U/L). No clinically significant change was observed in the serum levels of fasting blood glucose, AST, ALT and GGT, as well as in blood pressure and heart rate measures.

## Discussion

High cholesterol levels are a major risk factor for cardiovascular disorders and may require specific intervention with the prescription of an appropriate drug treatment [3] which has been shown to be able to reduce morbility and mortality in large cohorts of patients [28] and principally based on statins. Red yeast rice, obtained through rice fermentation by the fungus *Monascuspurpureus,* contains monacolin K a natural product chemically similar to lovastatin [29]. Monacolin K, similarly to synthetic statins, inhibits the activity of HMG-CoA (3-hydroxy-3-methyl-glutaryl coenzyme A) reductase that mediates the production of cholesterol synthesis. In this work we have investigated the lipid lowering capacities of a novel association containing monacolin K, L-arginine, coenzyme Q10 and ascorbic acid. L-arginine is an essential aminoacid with NO-mediated vasodilating properties [18–21], coenzyme Q has antioxidant activity [25] while ascorbic acid has antioxidant and vasoprotective activity [26, 27]. The effects of this association were compared with those of a commercially available dietary supplement containing monacolin K and coenzyme Q10. The study was conducted according to a rigorous randomized cross-over design. Our results were in agreement with previous research on monacolin K based dietary supplements and showed a statistically significant reduction of LDL-C levels. We were able to document a marked lowering effect of LDL-C (A: -40.1 mg/dL, − 25,6%; N: -36,4 mg/dL, − 23,3%), in line with previous reports [11, 13, 15, 30–32], with no difference in efficacy between the two formulations studied. In parallel with the reduction of LDL-C a decrease in Tot-C was documented for both formulations, and HDL-C was increased although reaching significant levels only with N. A significant reduction of triglycerides levels was found exclusively for the novel formulation tested (A). A clinically relevant effect of monacolin K-based supplements on triglycerides has been previously but not consistently reported [10], since this reduction was not documented for N we speculate that other compounds present in A, probably L-arginine, may have contributed to this effect [33].

Regarding side effects (adverse events) in our study 3/19 patients reported mild and transient gastrointestinal discomfort, and in 3/19 subjects elevation of CK occurred, with no associated myalgia. These figures are similar to what expected and already reported in the literature [34]. Among the limitations of this study we have to mention the open (not blinded) design of the study which was chosen because of the different nature of the pharmaceutical preparations of the compounds tested. The relatively low number of patients enrolled is an additional limitation, the impact of which was attenuated by the crossover design.

## Conclusions

Our results confirm the clinically meaningful lipid lowering properties of monacolin K and specifically the efficacy of a novel association of it with L-arginine, coenzyme Q10 and ascorbic acid. Furthermore, our results show a good safety profile of this association.

In addition to the very effective LDL-C lowering capacity it is worth noticing the significant reduction of triglycerides level achieved by A, which might be due to the presence of L-arginine in the formulation.

## Abbreviations

A: Argicolina
ALT: Alanine aminotransferase
AST: Aspartate aminotransferase
CK: Creatinekinase
CVDs: Cardiovascular diseases
GGT: Gamma-glutamyl-transpeptidase
HDL-C: HDL-cholesterol
HMG-CoA reductase: 3-hydroxy-3-methyl-glutaryl coenzyme A reductase
LDL-C: LDL-Cholesterol
N: Normolip 5
NO: Nitric oxide
TG: Triglycerides
Tot-C: Total cholesterol
